# Persistently high antibody responses after AS03-adjuvanted H1N1pdm09 vaccine: Dissecting the HA specific antibody response

**DOI:** 10.1038/s41541-021-00308-5

**Published:** 2021-04-01

**Authors:** Anders Madsen, Åsne Jul-Larsen, Mai-Chi Trieu, Florian Krammer, Rebecca J. Cox

**Affiliations:** 1 Influenza Centre, Department of Clinical Science, University of Bergen, Bergen, Norway; 2 Department of Microbiology, Icahn School of Medicine at Mount Sinai, New York, USA; 3 Department Microbiology, Haukeland University Hospital, Bergen, Norway

**Keywords:** Immunology, Vaccines, Inactivated vaccines, Vaccines, Inactivated vaccines

## Abstract

Current influenza vaccines have a suboptimal effectiveness. The introduction of a novel A/H1N1 influenza virus in 2009 (H1N1pdm09) provided a unique opportunity to study the humoral response to the AS03-adjuvanted H1N1pdm09 vaccine and repeated annual vaccination with the homologous virus in subsequent influenza seasons. Thirty-two HCWs immunized with the AS03-adjuvanted H1N1pdm09 vaccine in 2009 were divided into four groups based on the longevity of their antibody responses (persistently high or transient), and whether they were repeatedly annually vaccinated in the subsequent four influenza seasons or not. Serological assays were utilized to measure the quantity, quality and functionality of antibodies targeting the major surface glycoprotein hemagglutinin (HA). Persistent high responders (hemagglutination inhibition (HI) titre ≥ 80 at 12 months after H1N1pdm09 vaccination) had protective levels of HI antibodies throughout the study period. In addition, the quality and functionality of these antibodies were greater than the individuals who had a transient antibody response to the pandemic vaccine (HI titre < 40 at 12 months after H1N1pdm09 vaccination). All groups had similar levels of antibodies towards the conserved HA stalk domain. The level of HA head-specific antibodies gradually increased over time with annual vaccination in the transient responders. The AS03-adjuvanted H1N1pdm09 vaccine elicited a robust humoral response that persisted up to 5 years in some individuals. Seasonal annual vaccination boosted the HA-antibodies over time in individuals with a transient response to the pandemic H1N1pdm09 vaccine.

## Introduction

Influenza is a contagious respiratory pathogen that causes annual epidemics with an estimated 290,000–650,000 deaths each year^1^. Occasionally, influenza pandemics occur, causing a substantial burden for health care systems globally. Vaccination against influenza is the most effective measure to prevent infection and induces B cell responses leading to the production of neutralizing antibodies. While the licensed seasonal inactivated influenza vaccines (IIV) are effective, they lack two essential attributes; firstly the antibody response provide limited potential for cross-protection; and secondly, immunity appears to be of a short of duration^2^.

Vaccine-induced antibodies are predominantly directed to the major surface glycoprotein hemagglutinin (HA)^3–5^. The HA protein consists of a stalk domain and a head domain, the latter being the immunodominant part of the HA protein. Antibodies directed to the HA head domain prevent attachment of the virus to the sialic acids on host cells. HA head-specific antibodies can be detected with hemagglutination inhibition assay (HI), a serological assay commonly used for measuring vaccine immunogenicity. However, the HA head domain is particularly prone to mutations due to the continuous evolution of the virus in a process called antigenic drift. Thus, HA-head-specific antibodies generated in response to prior infection or immunizations are no longer neutralizing due to the antigenic plasticity of the influenza virus^6^. Antibodies targeting the conserved HA stalk domain are broadly cross-reactive and therefore the stalk is a candidate for novel broadly protective influenza vaccines^7^. Stalk-specific antibodies have other functions, including blocking viral fusion with the host cell and inducing natural killer (NK) cells and eliminating infected host cells through antibody-dependent cellular cytotoxicity (ADCC)^8^.

In 2009, a novel influenza A/H1N1 virus (H1N1pdm09) emerged, causing the first pandemic of the 21st century providing a unique opportunity to study the humoral response to a novel influenza virus. During the pandemic, healthcare workers (HCW) were prioritized for vaccination in order to maintain the integrity of the healthcare system and protect their patients. In Norway, a novel monovalent pandemic vaccine containing the H1N1pdm09 virus with an oil-in-water emulsion AS03-adjuvant was available in October 2009, just before the peak of the pandemic activity. The AS03 adjuvant improved the vaccine´s immunogenicity, allowing for dose sparing and increasing the number of available doses^9^. A 5-year cohort study of HCWs vaccinated with the AS03-adjuvanted H1N1pdm09 vaccine was conducted to investigate the antibody response to the AS03-adjuvanted H1N1pdm09 vaccine and subsequent annual vaccination containing the H1N1pdm09 virus^10^. Our initial results from the HCW cohort have shown that the AS03-adjuvanted vaccine provided robust durable antibody responses. However, the H1N1pdm09-specific antibody titres varied considerably between HCW one year after pandemic vaccination, ranging from protective levels in some individuals to undetectable in others. In order to better understand the longevity of the antibody responses, we dissected the HA-specific antibody response after the AS03-adjuvanted H1N1pdm09 vaccine with or without annual seasonal vaccination in the subsequent four influenza seasons.

## Results

### Study population

**Fig. 1:**
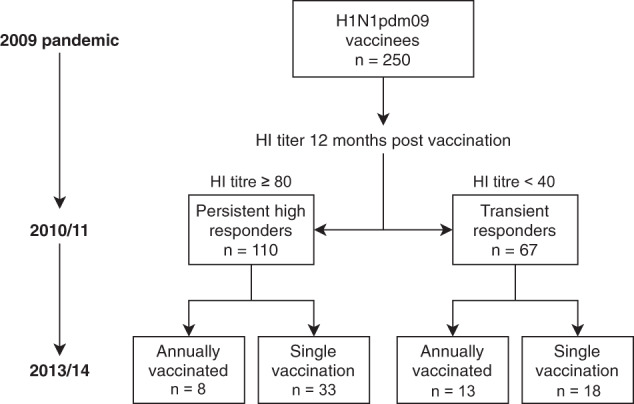
Study design. Healthcare workers (HCWs) were vaccinated in 2009 with the AS03-adjuvanted low-dose (3.75 μg HA) pandemic H1N1pdm09 vaccine (*N* = 250)^10^. Blood samples were collected prior to pandemic vaccination in 2009 and annually before the subsequent influenza seasons. HCWs were grouped based on their HI titres at 12 months after the adjuvanted pandemic H1N1pmd09 vaccine: those with HI ≥ 80 as “persistent high responders” and those with HI < 40 as “transient responders”. Subjects were further divided into subgroups based on their seasonal vaccination habits after pandemic vaccination: HCWs who were annually vaccinated in the subsequent four influenza seasons as “repeated vaccination group” and those who chose not to receive seasonal vaccination as “single vaccination group”. In this study, we included all eight individuals from the “Persistent high, repeated vaccination” group, and selected eight individuals that donated the most frequent blood samples in subsequent seasons from each of the three groups “Persistent high, single vaccination”, “Transient, repeated vaccination” and “Transient, single vaccination”.

A total of 32 HCWs were selected for the current study based on their HI response to pandemic H1N1pdm09 vaccine at 12 months post-vaccination and their vaccination habits during the subsequent influenza seasons. The HCWs were divided into four groups based on the longevity of their antibody responses (persistently high or transient), and whether they were repeatedly vaccinated in the subsequent four influenza seasons (repeated) or not (single) (Fig. 1). We analysed the durability and kinetics of the antibody responses after vaccination with AS03-adjuvanted H1N1pdm09 pandemic vaccine and subsequent seasonal vaccination. A majority of the HCWs in this study were female (81%), except for the group of single vaccinated persistent high responders, which had an even gender distribution (Table 1). This is similar to the gender distribution in the Norwegian health care sector. In total, at least 18 HCWs (56%) had previously been vaccinated with seasonal influenza vaccines before the 2009 H1N1 pandemic and most of them (14/18) belonged to the repeated vaccination groups. Of note, there were a higher proportion of individuals with pre-existing antibodies (HI ≥ 10) before pandemic vaccination among the persistent high responders (69%) compared to the transient responders (31%). This could be due to prior vaccination with IIVs before 2009 or working in a clinical department caring for infected patients^10,11^.

**Table 1:** Characteristics of study participants.

Characteristics	Persistent high responders		Transient responders	
	Single group	Repeated group	Single group	Repeated group
Average age at time of pandemic vaccination (range)	42 (26–67)	41 (34– 53)	37 (28–52)	50 (35– 62)
Previous seasonal influenza vaccination before 2009 (yes/no/unknown)	4/3/1	7/1/0	1/6/1	6/1/1
Seasonal vaccination in 2009 (yes/no/unknown)	1/7/0	1/6/1	1/7/0	1/7/0
Working department (non-clinical/clinical/infectious disease)	4/3/1	3/1/4	6/2/0	2/4/2
Detectable H1N1pdm09 antibodies (HI) at D0 (yes/no)	5/3	6/2	0/7	5/3
Underlying medical conditions (yes/no)	1/7	0/8	0/8	1/7

### Long-term maintenance of seroprotection in persistent high responders regardless of subsequent seasonal vaccination

**Fig. 2:**
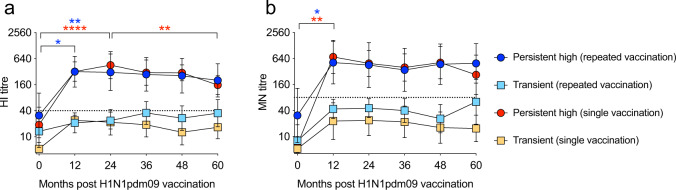
High antibody titres 12 months post H1N1pdm09 vaccination persisted up to four subsequent years, regardless of repeated seasonal influenza vaccination. The HI titres (**a**) and MN titres (**b**) against H1N1pdm09 virus were measured annually before each influenza season in persistent high responders (circle) and transient responders (square) after repeated vaccination (blue and turquoise) or single vaccination (red and orange). The geometric mean titres and 95% confidence intervals are presented. The dotted line at HI = 40 and MN = 80 represent a surrogate correlate of protection. Statistical differences between different time points were tested using two-way ANOVA with Tukey test for multiple comparisons. *P < 0.05, **P < 0.01, ****P < 0.0001.

The HI and neutralizing antibodies were assessed in all four groups of HCWs (Fig. 2 and Supplementary Table 1). The persistent high responders remained above the suggested thresholds of protection (HI ≥ 40 and MN ≥ 80) throughout the study period of 60 months, regardless of subsequent seasonal vaccination status^12,13^. However, we observed a significant decrease in HI titres between 24 and 60 months post-pandemic vaccination in the persistent high single vaccination group (*p* < 0.001) (Fig. 2a), although there were no significant differences between the repeatedly vaccinated and single vaccinated persistent high responders. Antibody-titres in the transient responders were significantly lower than the persistent high responders at 12 months post vaccination, and remained below the protective threshold (HI < 40, MN < 80) throughout 60 months, regardless of subsequent seasonal vaccination status. Using linear regression, we found that the transient responders who were repeatedly vaccinated had a gradual increase of HI and MN antibodies during the 60 months follow-up (HI: *p* < 0.05, *R*^2^ = 0.12. MN: *p* < 0.05, *R*^2^ = 0.10). In comparison, we observed a tendency of decreasing antibody titres in the transient responders who were not seasonally vaccinated.

### Comparable HA stalk-specific responses in persistent high and transient responders

**Fig. 3:**
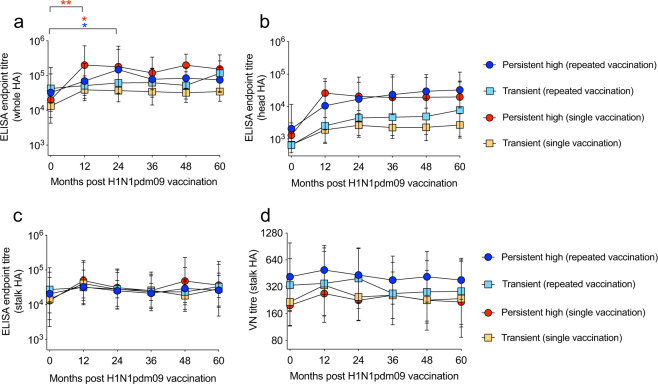
No differences in HA-stalk-specific antibodies between the persistent high and transient responders. The serum IgG binding to the HA protein of the H1N1pdm09 virus before each influenza season were measured by the ELISA assay. Persistent high responders are presented as circles and transient responders as squares. Repeated vaccination is shown in blue and turquoise, and single vaccination is shown in red and orange. The endpoint titres are shown for antibodies binding to different domains of the HA protein: whole HA (**a**), HA head (**b**) and HA stalk (**c**). Functional neutralizing HA-stalk specific antibodies were measured in the virus neutralization (VN) assay using a chimeric cH9/1N3 virus (**d**). Geometric mean titres and 95% confidence intervals are presented. Statistical differences between different time points were tested using two-way ANOVA with Tukey test for multiple comparisons. *P < 0.05, **P < 0.01.

The H1N1pdm09-specific antibody responses were further characterized in an ELISA to assess the composition of HA-reactive IgG (Fig. 3a–c and Supplementary Table 2). We found a tendency of higher levels of IgG towards the mutation-prone HA-head domain in the persistent high responders, compared to the transient responders (Fig. 3b). The HA-head reactive IgG increased with annual vaccination in the transient responders (linear regression: *p* < 0.05 and *R*^2^ = 0.09) and the persistent high repeated vaccination group (not significant). When assessing the HA stalk specific antibodies we used a chimeric cH9/1 HA, which has the H1 stalk domain and the H9 head domain. Since the subjects in this study are expected to be naïve to the avian H9, we assumed no or little cross-reactivity to the H9 head domain. We found that the level of IgG binding to the conserved HA-stalk domain remained similar between the groups (Fig. 3c). A further assessment of the functionality of the HA-stalk specific antibodies was conducted using the VN assay (Fig. 3d). Here, no significant differences between the groups were found, although we observed a trend of higher VN titres in the repeatedly vaccinated persistent high group throughout 60 months and the repeatedly vaccinated transient group at 0 and 24 months compared to the 2 single vaccinated groups. The HA-stalk-specific antibodies can also function through ADCC. We therefore performed an NK cell activation assay on four subjects from each HCW group who had similar levels HA stalk antibodies in ELISA (Supplementary Fig. 2). Here, we observed no significant increase in the ability to provide Fc-mediated protection after vaccination and no significant differences between the groups.

### Trend of higher avidity in persistent high responders

**Fig. 4:**
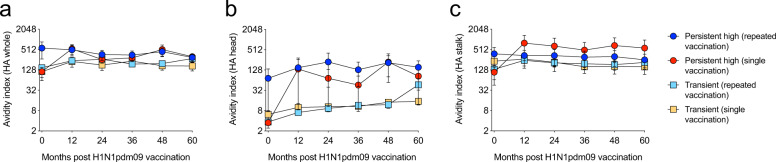
Trend of higher avidity in persistent high responders. The IgG avidity to the HA protein of the H1N1pdm09 virus before each influenza season were measured by the avidity ELISA assay against three different domains of the HA protein: whole HA (**a**), HA head (**b**) and HA stalk (**c**). Persistent high responders are shown as circles and transient responders as squares. Repeated vaccination is shown in blue and turquoise, and single vaccination is shown in red and orange. The means and standard errors of the means are shown.

To assess the quality of the HA-specific IgG in HCWs, we measured the avidity to the whole HA and the head and stalk domains separately (Fig. 4 and Supplementary Table 3). Pre-pandemic vaccination (day 0), the avidity to the whole HA and the HA-head was low in all groups except the persistent high repeated group, which had significantly higher avidities than the other groups (*p* < 0.05) (Fig. 4a, b). Overall, we found higher avidity towards the HA-stalk compared to the HA-head domain. There was a tendency of higher avidity in persistent high responders compared to the transient responders throughout the study period. However, this trend was less noticeable when measuring the avidity to the HA stalk and whole HA. In transient responders, the avidity index against HA-head significantly increased over time (repeated group; *p* < 0.05, *R*^2^ = 0.1 and single group; *p* < 0.01, *R*^2^ = 0.2).

## Discussion

Our study sought to better understand induction of persistently high antibody responses after pandemic vaccination with the AS03-adjuvanted H1N1pdm09 vaccine and the impact upon subsequent annual seasonal vaccination. There are several advantages in the use of AS03 adjuvant in a pandemic vaccine to prime the immune response. Firstly, the AS03-adjuvanted H1N1pmd09 vaccine lead to enhanced humoral responses compared to vaccination with a non-adjuvanted H1N1pdm09 vaccine^14,15^. Increasing vaccine immunogenicity is especially valuable in a pandemic scenario, where most of the population is immunologically naïve to the emerging virus. Specifically, the AS03 adjuvant functions by increasing antigen uptake and presentation in the local draining lymph nodes^9,16^. Furthermore, the adjuvant stimulates increased activation of naïve B-cell responses and increased adaptability of recalled memory B cells. This allowed dose sparing to 1/4 of the amount of antigen used in seasonal IIVs, which proved to be essential during the 2009 influenza pandemic to rapidly provide more vaccine doses from the available vaccine production.

Although the enhanced immunogenicity of the AS03-adjuvanted H1N1pdm09 led to robust antibody responses, there is a need for better understanding of the durability of antibody responses and the potential of annual vaccination to boost antibody responses. One of the main challenges for today’s IIVs is inducing long-lasting antibody responses that can provide protection against emerging influenza viruses. Traditionally, HI titres ≥ 40 are considered a correlate of protection in adults with >50% reduction in the risk of influenza infection^12^. In our study, we chose a 12-month interval as a measurement time point to get a better understanding of the baseline antibody levels before any subsequent seasonal vaccination and prior to circulation of the virus during the influenza season. Remarkably, we found that individuals with an HI titre ≥80 one year after the H1N1pdm09 pandemic vaccine maintained a persistently high antibody level throughout the 60 months study period, regardless of seasonal vaccination. These findings raise questions about whether there were other characteristics than their vaccination status or demographical characteristics that could explain their persistent antibody responses. Their demographic information such as age, working department or previous seasonal vaccination, could not explain the differences between the persistent high responders and transient responders. It is possible that the persistent high responders had a stronger germinal centre reaction to the H1N1pdm09 vaccine than the transient responders^17^. Since the persistent high responders had higher levels of H1N1pmd09 strain-specific antibodies, one could assume that the H1N1pdm09 vaccine was able to engage more naïve B cells in these individuals. The H1N1pdm09 vaccine may have stimulated more pre-existing memory B cells in the transient responders, targeting older H1N1 viruses resulting in an original antigenic sin effect. We therefore measured the HI response to previous circulating H1N1 viruses from 1977 to 2008 (Supplementary Fig. 1) pre- and post-H1N1pdm09 vaccination. However, we found that the transient responders had lower levels of back boosting to other H1N1 viruses compared to the persistent high responders. We could however see a higher proportion of individuals with pre-existing H1N1pdm09 antibodies prior to vaccination in the persistent high responders (69%) compared to the transient responders (31%). Similarly the percentage of individuals previously vaccinated with IIVs before 2009 was higher in the persistent high (69%) than the transient responders (44%). This suggests that a previous exposure to the H1N1pdm09 virus or previous seasonal vaccination was beneficial for achieving durable antibody responses to the H1N1pdm09 vaccine.

In addition to the HI and MN antibody responses, we have assessed the quality of antibodies and the level of antibodies targeting the conserved HA-stalk domain in our study. We found that despite lower HI and MN antibodies, the transient responders had similar levels of HA stalk-reactive antibodies compared to the persistent high responders. These HA stalk-reactive antibodies may have a critical role in protecting these individuals in the absence of head-specific HI antibodies. The HA stalk-specific antibodies are broadly cross-reactive and capable of preventing conformational changes in the HA, inhibiting viral egress and hindering cleavage activation of HA^7^. The antibody avidity is another important factor indicating the quality of antibodies. High avidity antibodies were associated with increased virus neutralization and milder disease upon infection with H1N1pdm09^18^. We found that the persistent high responders displayed the highest levels of antibody avidity throughout the study period.

Annual vaccination is recommended for all healthcare personnel in Norway to provide immunity against the frequently changing influenza viruses. In this cohort, the H1N1 antigen in the seasonal IIVs was the same as the antigen in the H1N1pdm09 vaccine throughout the study. Although the impact of annual homologous boosting is unclear, studies have found improved quality of B-cell and T-cell responses after repeated vaccination^19,20^. In our study, we observed a potential positive effect of repeated vaccination in the transient responders on the quantity (ELISA), quality (avidity) and functionality (MN) of the antibodies directed to the immunodominant HA head domain.

This study has limitations inherent in the long-term follow-up study design. Firstly, the size of the study population (*n* = 32) is relatively small. The strict inclusion criteria of having persistently high HI titres ≥80 at 12 months post-pandemic vaccination and annual seasonal vaccination in four subsequent seasons limited the study participants in the persistent high repeated vaccination group, thus affecting the number of participants in the other groups. Additionally, many individuals did not provide blood samples every year during the study, which restricted our selection of participants. A larger study is therefore needed to confirm our results. Secondly, the lack of virological and symptomatic surveillance of the HCWs means that we could not properly assess the influence of virus exposure and infection on the long-term antibody response. Furthermore, including additional sampling time points would give us a more comprehensive understanding of the serological responses after each vaccination. For future studies, assessing the immunoglobulin B-cell receptor repertoire and generating monoclonal antibodies from single-sorted B cells would provide valuable insight into the phenotypic, clonal and functional characteristics of the B-cell responses in the persistent high and transient responders.

In summary, the long-term antibody response of the persistently high responders differed from the transient responders in three different aspects with higher quantity, quality and functionality of influenza-specific antibodies in the persistent high responders compared to the transient responders. However, repeated annual vaccination with the same H1N1pdm09 antigen in the transient responders increased their H1N1pdm09-specific antibody repertoire over time. Our findings have implications for future development of influenza vaccines and the use of adjuvant.

## Methods

### Clinical study design

Two-hundred-and-fifty HCWs were vaccinated with the AS03-adjuvanted monovalent H1N1pdm09 pandemic influenza vaccine (Pandemrix, GlaxoSmithKline (GSK), Belgium) in 2009 and followed up for 5 years^10^ (www.Clinicaltrials.gov, NCT01003288). The study was approved by the local ethics committee (REKVest-2012/1772) and the Norwegian Medicines Agency. All participants provided written informed consent before inclusion. Blood samples were collected annually at 12 months intervals, prior to each seasonal influenza vaccination. We retrospectively grouped the HCWs based on their HI titres at 12 months post pandemic vaccination: HCW with HI titres ≥80 as “Persistent high responders” and those with HI titres <40 as “Transient responders”. We further divided the subjects into subgroups based on their vaccination habits after H1N1pdm09 pandemic vaccination: HCW who were annually vaccinated with seasonal vaccine during the subsequent four influenza seasons as “repeated vaccination group” and those who chose not to receive seasonal vaccination as “single vaccination group”. The seasonal influenza vaccines were non-adjuvanted split virus trivalent vaccines containing H1N1pdm09, A/H3N2 and B viruses. From the four groups “Persistent high, repeated vaccination” (*n* = 8), “Persistent high, single vaccination” (*n* = 33), “Transient, repeated vaccination” (*n* = 13) and “Transient, single vaccination” (*n* = 18), we included all eight individuals from the repeated vaccination group and chose eight individuals from each of other three groups that donated blood in most seasons to include in this study (Fig. 1). We excluded single vaccinated individuals who had a ≥4-fold increase in HI titre between any time points after 12 months post-pandemic H1N1pdm09 vaccination, as a sign of infection between the two time points.

### HA proteins and influenza viruses

Whole H1 HA (trimeric A/California/04/09) and chimeric cH9/1 (trimeric HA protein composed of a H1 stalk domain from A/Puerto Rico/8/1934 (H1N1) and an H9 head domain from A/guinea fowl/Hong Kong/WF10/99) were generated using the baculovirus expression system^21^. Recombinant baculoviruses were passaged three times through Sf9 cells, before infection of High-five cells. Purified proteins were analysed using sodium dodecyl sulfate polyacrylamide gel electrophoresis (SDS–PAGE) and quantified by infra-red spectrometer (DirectDetect^®^, Milipore Corporation). In addition, we used an influenza HA1 head (A/California/06/2009(H1N1)) hexahistidine-tagged protein (eEnzyme, Cat: IA-01SW-005P). Recombinant cH9/1N3 (for virus neutralization assay (VN)) and A/California/07/2009(H1N1) (for microneutralization assay (MN)) viruses were propagated in embryonated hen’s eggs 10 days after gestation. The A/California/07/2009(H1N1) virus was beta-propiolactone (BPL) inactivated for the HI assay.

### Hemagglutination inhibition assay

The HI assay measuring antibodies targeting the receptor-binding site of HA was performed as previously described^11^. In brief, eight HA units of the A/H1N1pdm09 strain (A/California/7/2009) and 0.7% turkey red blood cells were added to 2-fold serial dilutions of sera pre-treated with receptor destroying enzyme (Seiken, Japan). HI titres were defined as the reciprocal of the highest dilution of sera to prevent 50% agglutination. Undetectable HI titres <10 were assigned a value of 5 for calculation purposes.

### Microneutralization assay

The MN assay was performed as previously described using A/H1N1pdm09 like-virus (X179a)^22^. Briefly, sera were heat inactivated at 56 °C for 30 min and added in 2-fold serial dilutions to a 96-well plate (Nunc maxisorp™) with virus diluted to 2000 TCID_50_/ml (50% tissue culture infectious dose). Madin–Darby canine kidney (MDCK) cells were added after 1 h, and plates were further incubated for 16–18 h at 37 °C. Cells were subsequently fixed with hydrogen peroxide, and infected cells were detected by the presence of influenza A virus nucleoprotein. Mouse anti-influenza A virus nucleoprotein antibodies were added to the fixed cells and incubated for 1 h at 37 °C before adding horseradish peroxidase (HRP)-conjugated secondary antibody, and detected with a colorimetric substrate (3,30,5,50-3,30,5,50-tetramethylbenzidine (TMB)). MN titres were calculated as the dilution of serum at which 50% of MDCK cells were infected. Undetectable MN titres < 10 were assigned a value of 5 for calculation purposes.

### Indirect enzyme-linked immunosorbent assay (ELISA)

The amount of H1N1pdm09 HA-reactive serum IgG was determined by indirect ELISA as previously described^23^. In short, 96-well plates (Nunc maxisorp™) were coated with HA protein at a concentration of 1 μg/ml in PBS. The following H1N1pdm09 HA proteins were used: HA1 head (eEnzyme, Cat: IA-01SW-005P), whole HA and a chimeric stalk HA with an irrelevant H9 head domain (generated with baculovirus expression system). The plates were incubated at 4 °C overnight before adding 5-fold serial dilutions of sera. After 1 h incubation at 37 °C, HRP-conjugated monoclonal mouse anti-human IgG (BD Pharmingen™) was added to the HA-bound antibodies and detected by adding TMB substrate (BD Biosciences, USA). The endpoint titre was defined as the highest dilution of serum that gives a detectable measurement (an optical density (OD) ≥ 3 standard deviations above the mean of blank controls).

### Avidity ELISA

The avidity of the HA-reactive IgG was measured by avidity ELISA^23^. The procedure is similar to the indirect ELISA, with a few exceptions. Firstly, serum samples were diluted to an optical density (OD) of 0.7 ± 0.3 using an indirect ELISA. After 1 h incubation with sera, the plates were treated with 1.5 M chaotropic agent NaSCN (Sigma, St Louis, MO, USA). The avidity index was calculated as proportion of antibody remaining bound after treatment; (OD_Treated serum_/OD_Untreated serum_) multiplied by the dilution factor that was used on the serum sample to standardize the amount of IgG.

### Virus neutralization

Virus neutralization (VN) assay was preformed to detect HA stalk-specific antibodies inhibiting infection in cell culture^23^. In short, 2000 TCID_50_/ml of cH9/1N3 virus was incubated with 2-fold serial dilutions of heat-inactivated sera in viral growth medium (Dulbecco’s Modified Eagle’s Medium with tosyl phenylalanyl chloromethyl ketone-trypsin, 0.14% bovine serum albumin, 100 units/ml penicillin, 100 μg/ml streptomycin and 0.25 μg/ml amphotericin B) for 1 hour at 37 °C. The serum-virus mixture was added to a 96-well plate with confluent MDCK cells for 1 h at 37 °C. After incubation and washing to remove virus and sera, new medium with the same serum dilution was added to the MDCK-cells and incubated for 72 h. Subsequently, 50 μl of the supernatant was transferred to a 96-well V-bottom plate to measure the hemagglutination activity. The VN titre was defined as the highest dilution of serum causing 100% hemagglutination (using 0.7% human O^-^ blood).

### NK cell-mediated ADCC assay

The ADCC assay for measuring intracellular NK cell IFN-γ and CD107a expression was conducted and analysed with the gating strategy as previously described^23^. Briefly, 96-well plates were coated overnight at 4 °C with A/California/04/09 HA protein (1 μg/ml) and chimeric cH9/1 HA protein (1 μg/ml). The plates were then washed with PBS and incubated with heat-inactivated sera (prediluted 1:10) for 2 h at 37 °C. Plates were then washed again with PBS and incubated with 10^5^ CD16 176 v NK-92 cells per well (mycoplasma-free, human NK cell line expressing high affinity 176 V variant CD16 receptor) (Fox Chase Cancer Center, Philadelphia, PA, USA). As a negative control, NK-92 cells lacking the expression of CD16 were added to an additional well per sample. Cells were incubated with anti-CD107a-AF488 antibody (Biolegend, San Diego, CA, USA), Brefeldin A (5 μg/ml, BD) and monensin (5 μg/ml, BD) for 16 h at 37 °C. After incubation, the cells were stained with LIVE/DEAD Fixable Aqua dead cell staining kit (Invitrogen), anti-CD3-PE CF594 (BD) and anti-CD56-APC (BD) before intracellular staining with anti-IFN-γ-BV-421 (Biolegend). The cells were acquired on BD Fortessa. Data analysis was performed using FlowJo version 10 (treeStar).

### Statistical analysis

The statistical analysis was performed with GraphPad Prism version 7.0e for Mac, (GraphPad Software, USA). Statistical differences were tested using two-way ANOVA with Tukey test for multiple comparisons. Correlations are presented as Spearman *r*, alpha = 0.05. A correlation between antibody titres and years post H1N1pdm09 vaccination was identified using linear regression models. A *p*-value < 0.05 was considered statistically significant in all analyses.

### Reporting summary

Further information on research design is available in the Nature Research Reporting Summary linked to this article.

## Supplementary information

Supplementary Information

Reporting Summary

## Data Availability

The data sets generated during and/or analysed during the current study are available from the corresponding author on reasonable request.
