# Supplementary material for: Persistently high antibody responses after AS03-adjuvanted H1N1pdm09 vaccine: Dissecting the HA specific antibody response
Source: NPJ Vaccines. 2021 Apr 1;6:45. doi: 10.1038/s41541-021-00308-5 (PMC8016826; doi:10.1038/s41541-021-00308-5)
Supplement: Supplementary file 1 — Supplementary Information [file 41541_2021_308_MOESM1_ESM.pdf]

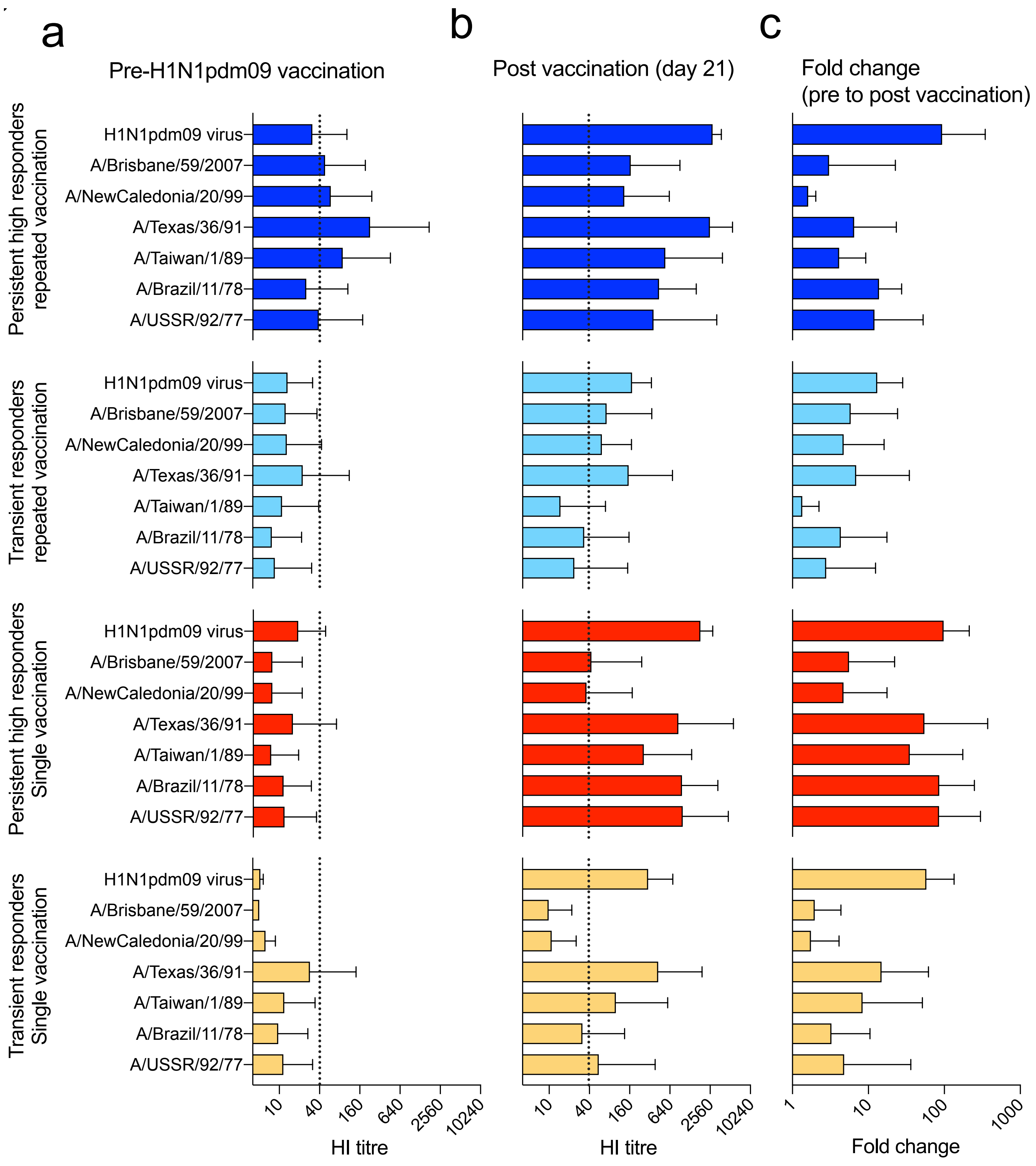

Supplementary Figure 1. Back-boosting to previous H1N1 viruses after H1N1pdm09 vaccination. The HI titres to six historical H1N1 vaccine strains and the H1N1pmd09 virus strain were measured pre-H1N1pdm09 vaccination (A) and at 21 days post-H1N1pdm09 vaccination (B). The dotted line at HI = 40 indicates a titre considered protective. C) The fold induction of HI titres between pre- and post-vaccination. Columns show geometric mean and error bars represent the upper 95% confidence interval.

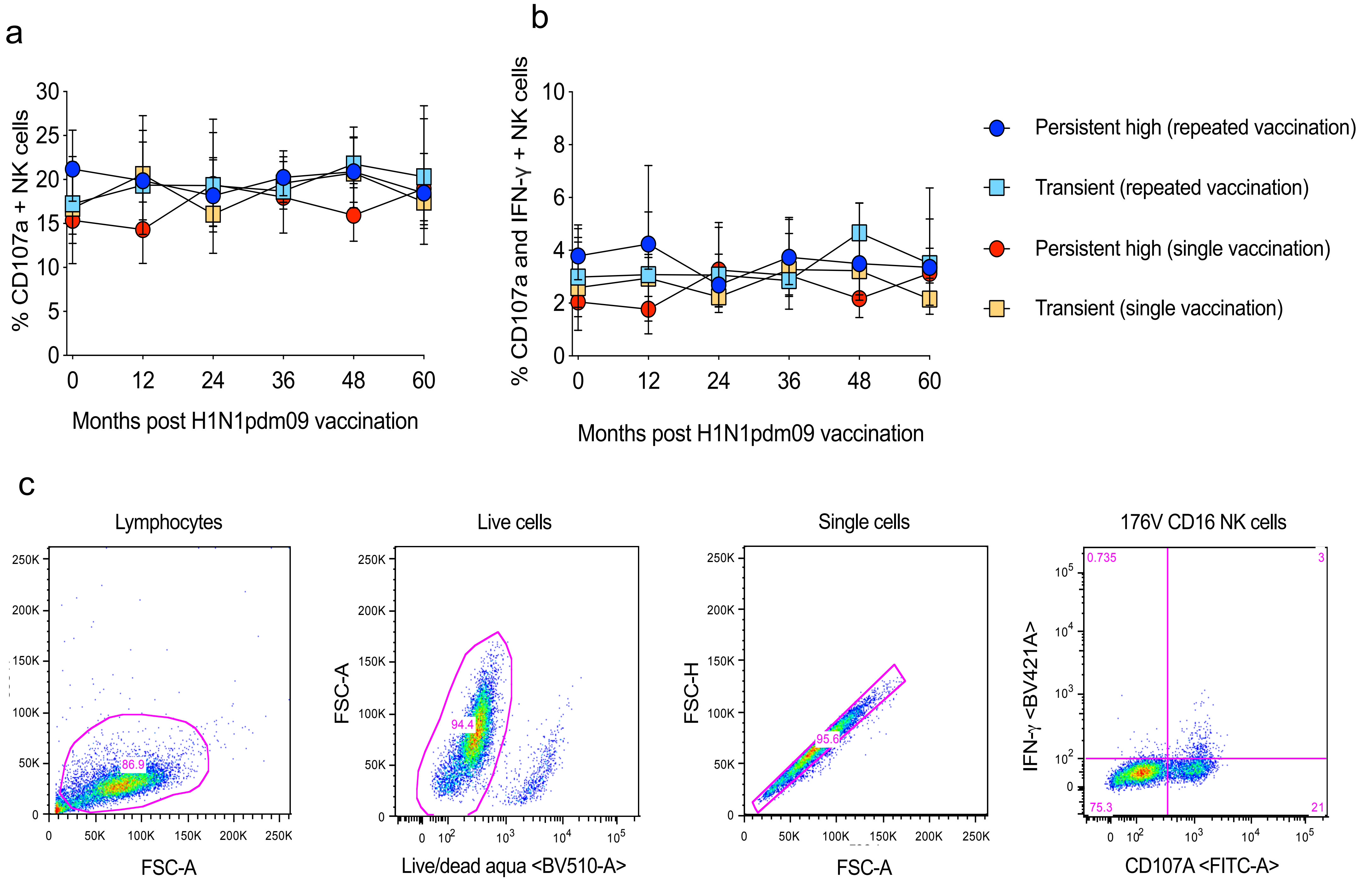

Supplementary Figure 2. No differences in FcγR-dependent NK cell activation between the HCW groups. The HA-specific antibodies capable of inducing ADCC were measured with NK-cell activation assay. Persistent high responders are presented in circles and transient responders in squares. Repeated vaccination is shown in blue and turquoise, and single vaccination is shown in red and orange. A) The frequency of NK cells expressing CD107a. B) The frequency of NK cells expressing CD107 and IFN-γ. The means and standard errors of the means are presented. C) Flow cytometry gating strategy.

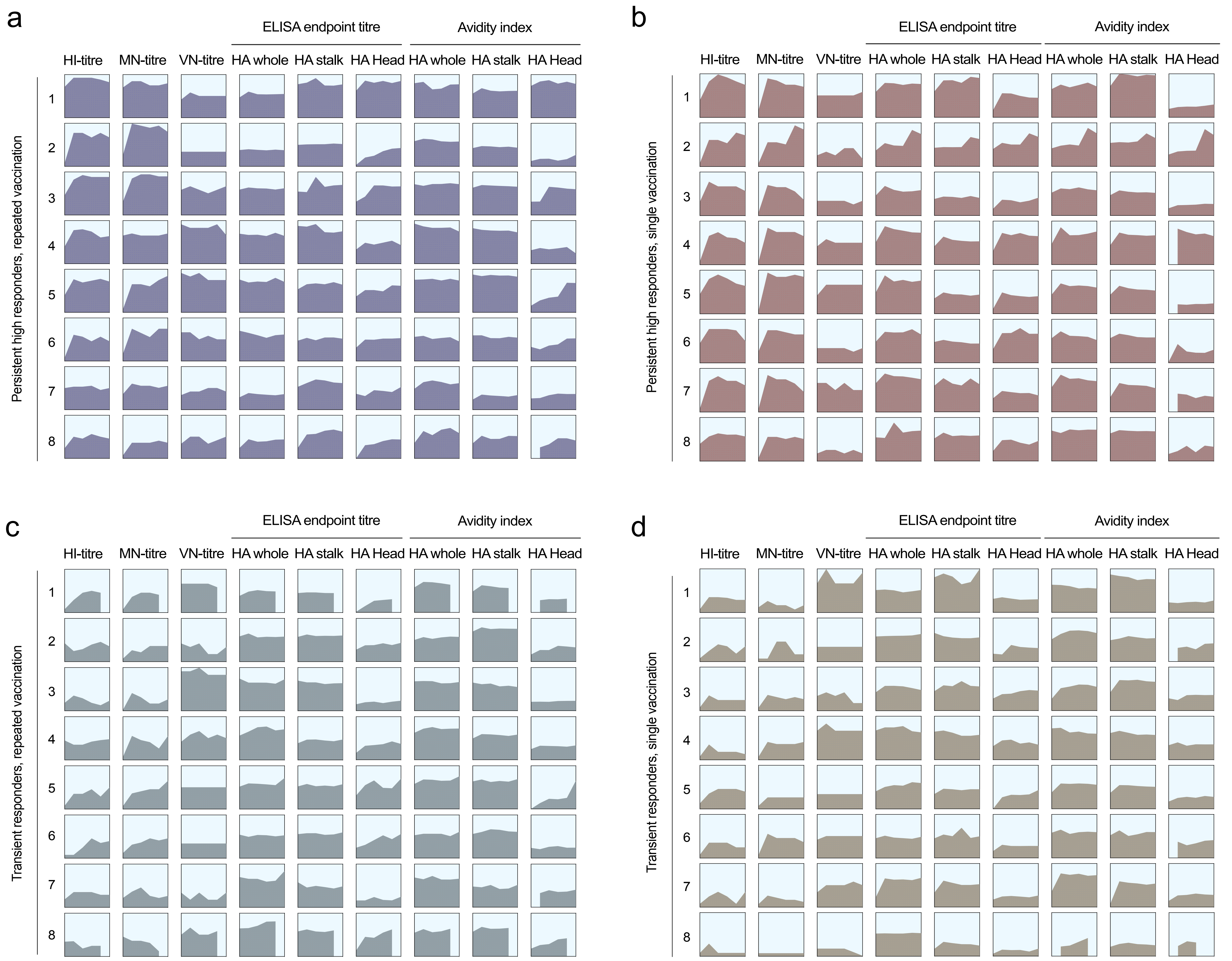

Supplementary Figure 3. An overview of the individual serological responses. The serological data is plotted for each individual in the "persistent high repeated vaccination" group (A), "persistent high single vaccination" group (B), "transient repeated vaccination" group (C), and "transient single vaccination" group (D). The X-axis represents the 60 months study period starting with the H1N1pdm09 pandemic vaccination in 2009. The scaling of the Y-axis remains the same between all individuals, but varies with the different assays. The 60-month serum sample for 2 subjects was not available (C). The missing values in the avidity columns are due to insufficient amounts HA-reactive IgG in the sample to meet the standardized criteria of the assay.

		Hemagglutination Inhibition titres						Microneutralization titres					
Group	Study subject	D0	12M	24M	36M	48M	60M	D0	12M	24M	36M	48M	60M
Persistent high, repeated vaccination	1	320	1280	1280	1280	960	640	453	1280	1280	640	640	905
	2	5	480	480	240	480	240	20	3582	2560	1810	2560	905
	3	60	640	1280	960	960	960	28	1280	2560	2560	1826	1826
	4	40	480	554	392	160	196	320	453	320	320	320	453
	5	40	453	277	365	480	320	5	320	320	226	640	1280
	6	5	160	120	60	120	60	10	640	320	160	640	640
	7	80	98	98	113	60	80	40	226	160	160	113	160
	8	14	80	60	120	80	60	5	40	40	40	57	40
Persistent high, single vaccination	1	40	640	1864	1191	659	392	10	1810	1280	640	640	453
	2	5	160	160	98	480	320	5	160	160	113	2560	1280
	3	24	480	240	240	240	120	5	453	320	320	160	40
	4	5	226	392	180	160	80	5	1280	640	640	640	320
	5	57	554	1076	596	277	196	5	2560	1280	1280	1810	1280
	6	57	480	480	480	392	80	20	640	640	453	320	320
	7	5	320	640	320	320	160	5	1280	640	640	320	80
	8	40	120	192	157	160	120	5	160	160	113	160	113
Transient, repeated vaccination	1	5	20	60	80	60		5	40	80	80	57	
	2	49	15	20	40	60	30	5	20	14	40	40	40
	3	10	30	20	10	7	14	4	57	28	10	10	20
	4	57	30	30	48	60	69	5	160	80	57	20	160
	5	5	30	30	60	20	80	5	40	57	80	80	320
	6	5	5	15	60	30	40	7	28	40	80	57	80
	7	10	30	30	30	20	20	14	40	80	20	14	20
	8	28	30	10	15	15		80	40	40	28	7	
Transient, single vaccination	1	5	30	30	27	20	20	7	20	10	10	5	10
	2	5	15	40	30	10	30	5	5	80	80	10	10
	3	5	30	15	15	15	15	5	40	28	20	28	20
	4	5	30	10	10	10	7	5	57	40	40	40	57
	5	7	30	60	60	60	40	5	20	20	20	20	20
	6	5	30	30	30	15	15	5	160	80	80	80	40
	7	5	15	30	15	5	30	5	20	14	10	10	7
	8	5	20	5	5	5	5	5	5	5	5	5	5

Supplementary Table 1: Individual serological HI responses from the cohort. An overview of the HCWs individual hemagglutination and microneutralization titres from pre-H1N1pdm09 vaccination (D0) to 60 months post pandemic vaccination from figure 2.

		ELISA Endpoint titre (whole HA)						ELISA Endpoint titre (head HA)					
Group	Study subject	D0	12M	24M	36M	48M	60M	D0	12M	24M	36M	48M	60M
Persistent high, repeated vaccination	1	302215	441378	1294385	220933	213704	292213	65023	712536	428133	660312	399189	659442
	2	17316	19269	19976	20184	23791	20834	143	829	1272	3814	6936	8134
	3	27571	24012	906530	85288	120560	136257	2500	8303	119833	119476	91837	94928
	4	847113	714409	1308284	188264	169303	232392	2978	15095	9786	15470	24564	8528
	5	25229	84272	103362	83546	137960	74005	4525	20659	20811	15417	64410	57943
	6	13201	25522	14528	28024	28448	21017	2500	16985	16347	21579	21795	24043
	7	24002	59415	139502	109833	67025	57724	4520	2625	10697	8999	6897	22837
	8	1243	30483	35182	74134	98289	58848	100	2346	3021	6296	9663	9149
Persistent high, single vaccination	1	42260	352707	319282	216655	299759	268688	538	30270	28406	15912	10860	10262
	2	4390	25482	14487	13214	575248	213764	6231	26452	17834	17658	253948	110563
	3	11633	111781	44202	28887	31530	41847	487	4215	3745	2285	3333	6769
	4	20134	904916	450962	315256	210402	192562	2563	182556	107400	177677	95173	84570
	5	15863	882862	210963	303247	192590	257118	305	15373	8152	6416	5358	6344
	6	15044	155157	133563	138338	288915	81675	2500	113256	116873	374306	97001	97798
	7	91108	892107	485259	450013	334743	232100	2500	14298	10826	8510	8842	5006
	8	27571	24012	906530	85288	120560	136257	1068	13952	15814	7652	4923	11584
Transient, repeated vaccination	1	5339	14790	21024	16548	15364		118	459	1608	1965	2341	
	2	41684	82045	32867	37931	35706	39839	1721	4894	5093	7870	5180	8768
	3	217987	83093	83298	81597	64238	185059	462	752	867	618	861	1088
	4	33522	85290	242842	288390	114454	148433	524	2875	3407	4202	8133	4472
	5	21160	45679	41882	36030	30130	167055	2500	29467	91593	15580	14011	140429
	6	27466	16324	25497	23918	18508	23597	1191	2353	7359	23040	9241	31688
	7	137026	89939	89268	45476	56996	582822	507	504	1142	577	516	1218
	8	80604	86634	150325	404982	429657		405	10591	8412	29107	62500	
Transient, single vaccination	1	20234	22882	19076	11832	15176	20407	2500	3601	2640	1993	2118	2242
	2	38229	41966	44430	45536	49272	69631	637	535	5053	3145	2702	2429
	3	7937	35365	36064	31878	21208	13477	1775	4963	5558	10015	13126	11409
	4	103763	214810	213669	290874	86912	66136	2500	9738	11592	4603	6109	3518
	5	6541	18355	27927	24444	55968	45634	100	1555	2797	2632	3074	8524
	6	10908	19724	12647	11066	9565	15215	507	2261	1713	1683	1726	1706
	7	1038	89917	66213	70435	59396	102686	595	1224	1377	1195	1002	1528
	8	21562	22536	21513	21609	25513	23564	179	467	419	434	300	592

		ELISA Endpoint titer (stalk HA)						Virusneutralization titres					
Group	Study subject	D0	12M	24M	36M	48M	60M	D0	12M	24M	36M	48M	60M
Persistent high, repeated vaccination	1	13025	51564	27457	26219	27627	29258	226	453	320	320	320	320
	2	4985	6042	5245	4672	5496	4807	160	160	160	160	160	160
	3	43824	73115	59994	56803	44572	60463	453	640	453	320	453	640
	4	131255	97763	103266	75757	162146	77321	1810	1280	1280	1280	1810	640
	5	258269	128382	160714	143811	386302	148103	1810	1280	1810	905	905	905
	6	139886	84741	48554	25719	48863	57173	640	640	320	453	320	320
	7	1954	5402	4226	3598	3164	4194	160	226	226	320	320	226
	8	1238	9707	5389	5953	8789	9285	160	320	320	160	226	320
Persistent high, single vaccination	1	45857	626191	658025	356024	1468631	1109798	320	320	320	320	320	453
	2	8279	9760	9516	9753	105252	65044	113	160	113	226	226	80
	3	5068	8872	8231	6499	10112	6497	160	160	160	160	113	160
	4	7718	75577	35438	27798	24180	24980	226	453	320	320	320	320
	5	3783	15477	10001	9569	7163	9363	226	640	640	640	640	640
	6	13994	22801	15247	13101	9522	9645	160	160	160	160	113	160
	7	50452	296118	81658	52614	275595	68236	640	640	320	640	320	320
	8	21244	82717	37119	42775	38527	43023	80	113	113	80	113	80
Transient, repeated vaccination	1	10509	12271	12128	10529	10377		640	640	640	640	453	
	2	37083	65119	45450	48122	46829	51938	226	160	226	80	80	160
	3	134238	88250	88230	59071	69092	72839	1810	1810	2560	1280	1280	1280
	4	5429	12665	13231	10319	8699	13094	226	453	640	320	453	320
	5	22361	39708	23219	27091	22666	37602	320	320	320	320	320	320
	6	31271	38725	38929	26229	29053	48115	160	160	160	160	160	160
	7	39867	11372	16640	12556	8984	15087	160	80	160	80	80	160
	8	39296	62889	38665	34257	49969		320	640	320	320	453	
Transient, single vaccination	1	415902	1003590	487643	78572	131584	2949543	640	2560	640	640	640	1810
	2	87694	40312	29070	25075	24941	30307	160	160	160	160	160	160
	3	9999	40079	32622	108222	35064	30623	160	226	160	226	80	80
	4	77701	101026	60512	29200	29105	36683	640	1280	640	640	640	640
	5	3371	11730	10337	8224	10363	10361	160	160	160	160	160	160
	6	10348	25522	17283	126659	11397	16830	226	320	320	320	320	320
	7	3056	51780	30611	18795	18840	22516	160	320	320	320	453	320
	8	570	2951	2190	1716	1685	1220	80	80	80	80	57	40

Supplementary Table 2: Individual serological ELISA responses from the cohort. An overview of the HCWs individual ELISA IgG endpoint titres against the whole, head and stalk hemagglutnin (HA), and the virus neutralization titres from figure 3.

		Avidity index (whole HA)						Avidity index (head HA)					
Group	Study subject	D0	12M	24M	36M	48M	60M	D0	12M	24M	36M	48M	60M
Persistent high, repeated vaccination	1	800	1055	226	264	609	640	464	1139	1486	737	1093	692
	2	119	193	171	115	104	110	2	3	3	2	3	7
	3	377	270	392	396	464	378	10	11	222	190	152	135
	4	2534	1430	1060	1067	1146	490	11	17	13	16	20	6
	5	545	583	661	470	665	458	3	8	14	17	295	278
	6	96	119	62	76	89	37	12	7	16	20	67	71
	7	49	192	272	197	123	157	7	7	13	18	17	17
	8	16	184	79	243	347	117		6	12	42	41	26
Persistent high, single vaccination	1	209	521	300	472	780	353	4	5	6	6	7	9
	2	26	45	58	47	1691	552	7	14	16	16	1337	397
	3	70	164	142	203	137	113	3	5	6	6	7	7
	4	44	1344	263	271	364	570		1004	507	315	371	219
	5	32	318	183	208	186	135		4	4	5	5	5
	6	100	405	163	151	131	68	1	30	7	5	5	10
	7	99	1300	564	627	415	350		27	22	11	18	14
	8	329	199	412	350	397	390	3	5	15	4	15	12
Transient, repeated vaccination	1	113	351	327	261	197			8	10	10	11	
	2	56	101	71	95	112	105	3	7	7	17	15	12
	3	279	295	287	183	190	240	4	4	4	5	5	5
	4	172	400	558	368	400	411	6	12	11	11	10	13
	5	113	284	268	216	226	542	1	2	5	5	6	221
	6	72	98	98	97	60	145	5	4	7	8	6	6
	7	299	208	407	197	195	233		12	22	15	16	24
	8	85	178	94	115	192		3	7	8	22	27	
Transient, single vaccination	1	187	176	139	88	98	86	5	4	5	6	5	8
	2	62	183	354	375	326	216		11	14	8	24	27
	3	7	64	89	135	128	84	8	6	15	16	16	16
	4	394	442	170	205	140	133	13	23	11	15	15	15
	5	19	115	107	116	108	89	3	6	8	6	9	8
	6	113	209	89	127	139	126		18	9	13	22	25
	7	16	627	475	584	389	438	2	7	8	10	9	8
	8		5	6		27			5	14	11		

		Avidity index (stalk HA)					
Group	Study subject	D0	12M	24M	36M	48M	60M
Persistent high, repeated vaccination	1	95	285	172	150	163	168
	2	28	43	40	35	38	31
	3	184	318	288	266	249	225
	4	994	706	616	588	602	419
	5	1607	1159	1472	1285	1310	1023
	6	130	133	74	73	97	82
	7	5	15	12	11	10	13
	8	15	70	36	37	68	65
Persistent high, single vaccination	1	385	4669	3929	2839	3887	3453
	2	80	93	86	108	556	212
	3	42	111	84	73	76	67
	4	35	452	291	270	237	258
	5	32	195	119	92	88	74
	6	103	249	185	125	117	117
	7	13	238	160	150	134	83
	8	185	358	312	288	296	280
Transient, repeated vaccination	1	49	176	143	108	100	
	2	319	754	533	588	570	557
	3	215	153	175	98	112	86
	4	53	131	124	108	90	119
	5	191	285	169	208	181	305
	6	95	141	246	220	160	145
	7	47	40	64	28	31	65
	8	103	282	190	197	226	
Transient, single vaccination	1	1526	1100	888	479	592	571
	2	51	71	117	91	72	78
	3	28	335	316	354	263	234
	4	154	238	160	105	113	103
	5	17	83	81	69	62	59
	6	68	182	59	82	131	128
	7	1	124	94	81	63	79
	8	4	8	10	8	7	6

Supplementary Table 3: Individual avidity responses from the cohort. An overview of the HCWs individual IgG avidity index against the whole, head and stalk hemagglutinin (HA) from figure 4.
